# Participatory study of medicinal plants used in the control of gastrointestinal parasites in donkeys in Eastern Shewa and Arsi zones of Oromia region, Ethiopia

**DOI:** 10.1186/1746-6148-9-179

**Published:** 2013-09-11

**Authors:** Claire E Scantlebury, Laura Peachey, Jane Hodgkinson, Jacqui B Matthews, Andrew Trawford, Getachew Mulugeta, Gebre Tefera, Gina L Pinchbeck

**Affiliations:** 1 Institute of Infection and Global Health and School of Veterinary Science, University of Liverpool, Leahurst Campus, Cheshire, UK; 2 Moredun Research Institute, Edinburgh EH26 0PZ, UK; 3 The Donkey Sanctuary, Sidmouth, Devon, UK; 4 College of Veterinary Medicine and Agriculture, Addis Ababa University, Debre Zeyit, P.O. Box 34, Ethiopia

**Keywords:** Ethnoveterinary, Anthelmintic, Nematode, Participatory, Donkey, Equid, Ethiopia, Thematic analysis

## Abstract

**Background:**

Gastrointestinal nematode infections constitute a threat to the health and welfare of donkeys worldwide. Their primary means of control is via anthelmintic treatments; however, use of these drugs has constraints in developing countries, including cost, limited availability, access to cheaper generic forms of variable quality and potential anthelmintic resistance. As an alternative, bioactive plants have been proposed as an option to treat and control gastrointestinal helminths in donkeys. This study aimed to use participatory methodology to explore donkey owner knowledge, attitudes and beliefs relating to the use of plant-based treatments for gastrointestinal parasites of donkeys in Ethiopia.

**Results:**

In focus groups, 22/29 groups stated they knew of plants used for the treatment of gastrointestinal parasites in donkeys. All groups volunteered plants that were used in cattle and/or small ruminants. In total, 21 plants were named by participants. ‘Koso’ (*Hagenia abyssinica*) ‘Grawa’ (*Vernonia amygdalina*) and a mixed roots and leaves preparation were the most frequently named plant preparations. ‘Enkoko’ *(Embelia shimperi*) and ‘a mixture of roots and leaves’ were ranked highly for effectiveness in donkeys. However, ‘Grawa’ and ‘Koso’ were the highest ranked when taking into account both the rank position and the number of groups ranking the plant.

Thematic analysis of participants’ current attitudes and beliefs surrounding traditional plant-based remedies for gastrointestinal parasites revealed that anthelmintics obtained from clinics were generally favoured due to their ease of administration and perceived higher effectiveness. There was doubt surrounding the effectiveness of some plant-based treatments, but there were also perceived advantages including their low cost, ease of cultivation and availability. However, plant-based treatments were considered a “*past trend”* and people favoured “*modern”* medicine, particularly among the younger generation.

**Conclusions:**

There was extensive knowledge of plant-based treatments for gastrointestinal parasites in livestock in Ethiopia. In donkeys, Koso (*Hagenia abyssinica*), Grawa (*Vernonia amygdalina*), Enkoko (*Embelia shimperi*) and ‘mixed roots and leaves’ were the most frequently named and/or highest ranked plants with reported efficacy against gastrointestinal parasites. Further *in vitro* and *in vivo* investigation of these plants is now required to determine viable alternatives for the treatment and control of gastrointestinal parasites in Ethiopia.

## Background

Gastrointestinal nematode infections constitute a major threat to the health and welfare of donkeys worldwide. The strongyle nematode species, in particular the cyathostomins, are the most numerous and pathogenic parasites of equids both in the UK and in developing countries [1,2]. These parasites can be responsible for considerable morbidity and mortality in horses [2,3] and may have negative effects on performance and productivity in donkeys [4,5]. Helminthiasis has been documented as a significant problem in working equids, many having polyparasitism [1,6-8]. There is a high prevalence and, often, high infection intensities in donkeys [1,9], making this a significant health concern in Ethiopia. As most animals do not acquire 100% immunity to intestinal nematodes [10], there is a need for life-long control strategies to reduce the burden of infection, particularly in those individuals who remain susceptible to high levels of infection throughout their lives [3].

The currently available anthelmintics (benzimidazoles, tetrahydropyrimidines and macrocyclic lactones) have been widely used against equine gastrointestinal helminths prophylactically and chemotherapeutically for many years. However, anthelmintic resistance is thought to be present in many populations and threatens sustainable control in future [3,11]. Further, in some developing countries, where donkeys are relied upon for transportation, there may be other constraints to the use of manufactured anthelmintics, such as limited availability and excessive cost. Anthelmintics may be diluted before being sold or may be used at incorrect dose rates, which may further accelerate the development of resistance in these populations [12]. Hence, there is a need to explore alternative methods of control of gastrointestinal helminths in donkeys in these parts of the world.

According to circumstances and depending on their relative efficacy, bioactive plants with anthelmintic properties offer an alternative that may overcome some of these problems [13]. Ethnoveterinary medicines (including bioactive plants) have been used for centuries for the treatment of a variety of health problems in humans and animals and it is estimated that up to 80% of Africa’s population use traditional medicine for their health needs [14]. A number of studies from Ethiopia and elsewhere have reported plants that are believed to have efficacy against internal parasites in ruminants, chickens or people [15-23]; however, none have identified plant-based anthelmintics specifically for use in donkeys or other equids.

In Ethiopia, horses and donkeys play a crucial role in both urban and rural communities, where they are used to transport a variety of goods including crops, firewood, household consumables and water [24]. The UN Food and Agriculture Organisation estimate that there are over 7 million donkeys, mules and horses in Ethiopia [25], and it has the second largest donkey population in the world. Added to this, there is a rich diversity of plant species among Ethiopia’s varied topography [26], generating a long tradition of the use of plants for medicinal purposes. This study aimed to use participatory approaches [27] to explore donkey owner knowledge, attitudes and beliefs relating to the use of plant-based treatments for gastrointestinal parasites in these animals in Ethiopia. In addition, owner recognition of gastrointestinal parasitic disease was explored.

## Results

In total, 182 donkey owners participated in 29 focus group discussions. The majority of these (94%) were male participants ranging in age from >16 to <80 years.

### Participant reported signs of gastrointestinal parasites

In cattle, small ruminants and donkeys, the most frequently named sign recognised as indicative of the presence of gastrointestinal parasites was observing worms in faeces (Table 1), followed by loss of body condition. In all groups, a combination of signs were volunteered, possibly indicating that a number of signs are recognised and assessed together before an animal is regarded as having gastrointestinal parasites. The local term used for internal parasites in animals was ‘Maga’. In donkeys, two types of worms were described as being present in the faeces, which were long white worms and ‘alive’ red worms.

**Table 1:** Signs of gastrointestinal parasites in donkeys and ruminants reported by 29 groups of donkey owners

**Signs attributed to gastrointestinal parasites**	**Donkey n groups (%)**	**Cattle n groups (%)**	**Sheep and goats n groups (%)**
Worms in faeces	28 (96.6)	25 (86.2)	20 (69.0)
Loss of body condition	25 (86.2)	22 (75.9)	10 (34.5)
Colic	3 (10.3)	5 (17.2)	3 (10.3)
Bloat	5 (17.2)	4 (13.8)	2 (6.9)
Rough hair coat / loss of hair	5 (17.2)	3 (10.3)	1 (3.4)
Cough	11 (37.9)	4 (13.8)	8 (27.6)
Loss of appetite	9 (31.0)	8 (27.6)	1 (3.4)
Diarrhoea	-	14 (48.3)	5 (17.2)
Gut sounds	4 (13.8)	-	2 (6.9)
Eggs on mane	2 (6.9)	-	-
Lice / external parasites	-	2 (6.9)	3 (10.3)
Bottle jaw	-	5 (17.2)	7 (24.1)
Cysts (internal organs)	-	-	1 (3.4)
Pungent smell faeces	-	2 (6.9)	-
Bloody urine	-	2 (6.9)	-
Miscellaneous	8 (27.6)	4 (13.8)	-

### Plant-based treatments for gastrointestinal parasites

All groups volunteered plants that were used as anthelmintics in cattle and/or small ruminants, but only 22/29 groups stated they knew of plants for use in donkeys. Table 2 summarises all the plants named in the focus groups believed to be efficacious against gastrointestinal parasites for use in donkeys, cattle and small ruminants. ‘Koso’ (*Hagenia abyssinica*) ‘Grawa’ (*Vernonia amygdalina*) and ‘mixed roots and leaves’ were the most frequently named plant preparations. The unnamed ‘mixtures of roots / leaves’ varied considerably and were often recipes handed down through the generations that were prepared and sold for use by local villagers. The plant ranking data for donkeys (Table 3) shows that although ‘Enkoko’ *(Embelia schimperi)*, ‘mixture of roots and leaves’ and ‘Abdul salim’ (unknown) had a high average rank score, ‘Grawa’ and ‘Koso’ were the highest ranked when taking into account both the rank position and the number of groups ranking the plant (reported as combined rank score). The informant consensus (calculated at group level in this study) was 0.621. There was considerable variation in the described modes of preparation; these included techniques such as crushing, drying, mixing with water or salt, fermentation and smoking (inhalation). Aside from the group of remedies presented as ‘mixed roots / leaves’, 10 plants were prepared by crushing and infusion in water, 5 were infused with water with or without salt or other plants, 2 were prepared as concoctions, 1 crushed, 1 seeds of the plant were mixed with salt, 1 was in a powder form dissolved in water and 1 was smoked. Plant preparations were often administered by drenching the donkey using a glass bottle (for example ‘Coca-Cola’™ bottle). Additionally, dosage information was difficult to obtain, with estimations of amounts or volumes being illustrated in measures such as ‘handfuls’ for leaves, ‘finger lengths’ for roots and drinks bottles for liquids. A range of side effects were reported and these were sometimes attributed to variation in preparation methods and dose (Table 2).

**Table 2:** Plants used as anthelmintics in donkeys and ruminants volunteered by 29 focus groups (made up of 182 individuals)

**Scientific name**	**Traditional name**	**Frequency of groups (%) volunteering plants for use in donkeys**	**Frequency of groups (%) volunteering plants for use in cattle and/or small ruminants**	**Reported potential side effects**
*Hagenia abyssinica* (Bruce) J.F. Gmel	Koso (Am)	9 (31)	9 (31)	Diarrhoea can kill if overdose
Unknown	Mixed roots / leaves / traditional remedy	8(27.6)	12 (41.4)	
*Vernonia amygdalina* Delile	Grawa (Am)	7 (24.1)	13 (44.8)	Diarrhoea can kill if overdose
*Embelia schimperi* Vatke	Enkoko (Am) / Hanko (Or)	4 (13.8)	0	Abdominal pain and diarrhoea, bitter taste
*Cucumis prophetarum* C.A. Mey ex. Cogn	Holoto (Or)	3 (10.3)	2 (6.9)	Severe diarrhoea and can kill if overdose
*Verbascum sinaiticum* Benth.	Gura Harre (Or) / Yeahiya joro (Am)Donkey ear	3 (10.3)	1 (3.4)	None reported
*Withania somnifera* (L.) Dunal	Wahale (Or) / Gizawa (Am)	3 (10.3)	1 (3.4)	None reported
*Tapinanthus globiferus* Tiegh.	Harmuu (Or)(parasitic plant)	1 (3.4)	0	None if correct dose
*Phytolacca dodecandra* L’Hér	Endod (Am) / Handode (Or)	1 (3.4)	0	Acidic effect in stomach, can create burning sensation
Unknown	Yeare Geleba (Am) / Geleba atara (Or) Bean Straw	1 (3.4)	0	
*Trigonella foenum-graecum* L.	Abish (Am)	1 (3.4)	2 (6.9)	None reported
Unknown ‘root’	Buri (Or)	1 (3.4)	0	Severe diarrhoea and can kill if overdose
Unknown	Abdul Salim	1 (3.4)	1 (3.4)	Bitter taste if overdose can kill
*Dodonaea angustifolia* L.F.	Kitkita (Am)	1 (3.4)	0	Severe diarrhoea and can kill
Unknown	Sara-aja (Or)	1 (3.4)	2 (6.9)	None reported
*Croton macrostachyus* Hochst. ex. Delile	Bisana (Am)	1 (3.4)	0	Burning sensation and severe diarrhoea
Unknown	Chobi (Or)	0	1 (3.4)	None if use root, juice from plant is irritant to skin
Unknown	Feto (Or/Am)	0	1 (3.4)	None reported
*Nicotiana tabacum* L.	Tobacco	0	10 (34.5%)	Diarrhoea if overdose unconsciousness and can kill
*Melia azedarach* L.	Milia	0	1 (3.4)	None reported
*Capparis cartilaginea* Decne.	Delensisa (Or)	0	2 (6.9)	Diarrhoea overdose can kill
Unknown	Keskesae (Am)	0	1 (3.4)	None reported

NB: International plant name index (IPNI) names including latin and author names for each plant species are given where available.

Where a plant is labelled ‘unknown’, we were unable to obtain a voucher specimen that was positively identified by a respondent either due to season or locality.

Where known, the language for each traditional name given is noted as either Or (Oromic) or Am (Amharic).

**Table 3:** Plant rankings for perceived effectiveness in donkeys (results from 29 focus groups)

	***Vernonia amygdalina *(Delile) (Grawa)**	***Hagenia abyssinica *(Bruce) J.F. Gmel (Koso)**	***Cucumis prophetarum *(C.A. Mey ex. Cogn) (Holoto)**	***Embelia schimperi *(Vatke) (Enkoko)**	***Withania somnifera *(L. Dunal) (Wahale)**	**Roots and leaves mixture**	**Abdul Salim**	**Roots of plant mix**	***Tapinanthus globiferus *(Harmuu) (parasitic plant)**	***Verbascum sinaiticum *(Benth) (Gura Harre)**	***Trigonella foenum-graecum *(Abish)**	***Phytolacca dodecandra *(Sesse & Moc.) (Endod)**
Average rank score	2.2	2	3	1	2.5	1	1	2	2	2	3	3
Combined rank score	27	23	14	8	5	4	4	3	3	3	2	2
Number of groups volunteered this plant	6	6	4	2	2	1	1	1	1	1	1	1

### Attitudes towards ‘pharmaceutical’ and ‘traditional’ medicine

Table 4 details the super-ordinate (or key) themes and examples of sub-themes within each category. It was apparent that, although plant-based ‘traditional’ medicines were familiar to the current generation, pharmaceutical anthelmintic preparations obtained from the animal health clinics were generally favoured. Numerous reasons were cited; for example, pharmaceutical preparations derived from clinics were widely available at all study sites and were perceived to be more *“modern”* and even *“civilised”* making them a more attractive choice*.* This was because “*professional people”*, whose advice was held in high regard, prescribed them and there was also the reassurance that the products had been tested experimentally. Additionally, it was reported that these professionals (referring to local development agents/doctors/veterinarians/pharmacists/animal health workers) advised against the use of ‘traditional’ medicines due to the risk of side effects, uncertainty surrounding correct dosing and risks associated with drenching donkeys.

…“Whenever there is any problem with their animal they will take (the animal) to the clinic and the first question by the professionals is ‘have they given any traditional medicine to this animal’, such a question is not good for us they said so they have already stopped giving any traditional medicine to these animals because the animal health professionals do not advise them to use”…

**Table 4:** Summaries of superordinate themes from thematic analysis of discussion surrounding anthelmintic strategies in donkeys and other species

**Superordinate (key) themes**	**Example sub-themes**
Attitudes to medicines from the clinic	Preferences for either clinic or traditional plant based medicine.
	Clinic medicines are perceived as more modern, professional or scientific.
Attitudes and beliefs surrounding traditional plant based medicine	Preferences for either traditional or clinic medicines.
	Significance of societal influences upon these preferences.
	Traditional plant based medicines considered a ’past trend’.
	Interpretation of the response to plant based medicines within the animal as indicative of strength / efficacy and reported side effects.
	Spiritual connections and plant based medicine.
Origins of traditional plant based medicine	Evolved from cattle preparations.
	Inherited knowledge from fathers, passed down through the generations.
	Religious texts.
Beliefs of when to worm donkeys	Interpretation of clinical signs as gastrointestinal parasites.
	Selection of individuals or group to de-worm.
	Frequency of worming.
	Sources of advice.
Other non-plant based preparations for de-worming	Including: fermented butter, lake water, rotten egg, oil seed, alcohol.

There was additional confidence in pharmaceutical anthelmintic preparations as they were prescribed after specific diagnosis by a clinician, whereas ‘traditional’ medicines were less specific, often with the same plant preparation used for a range of different problems. Pharmaceutical preparations were reportedly easy to administer and effective after one treatment whereas plant-based ‘traditional’ medicines often required multiple or prolonged dosing before any efficacy was observed along with a risk of side effects. Practical problems with administering plant-based treatments also made them less favourable, as often it required drenching the animal with large volumes of fluid.

Reported disadvantages of pharmaceutical preparations were the, albeit, small number of observed side effects. Nevertheless, there were some positive reports in favour of plant-based ‘traditional’ medicines including, their low cost (even no cost in some cases), ease of cultivating at home and availability where no clinics were accessible. One traditional healer described villagers coming to him in the night for his plant remedies. However, in some areas, the reduction in demand for specific plant-based preparations meant that they were no longer available at the market. One participant voiced concern about deforestation in some areas resulting in difficulties sourcing some plant species.

In some instances plant-based ‘traditional’ medicines were believed to be more effective than pharmaceutical preparations; for example, some people considered ‘Koso’ to be a superior treatment, particularly in people. However, the reported side effects were a major consideration and ranged from diarrhoea to death if overdosed.

…“‘koso’ works better than a tablet but the only problem is the side effect, that is why they prefer to use tablet even though Koso is more effective”…

In some cases plant-based treatments for donkeys had developed from treatments that were previously used in cattle or sheep and goats.

…“When they do not have any option, they use it as an option for donkeys, they assume it will probably treat donkeys but they are not sure”…

One of the key questions asked whether people would be likely to return to plant-based treatments for wormers. Responses were orientated towards a preference for scientific justification, including approval by professionals and scientific testing. Additionally if the plant could be grown at home, this would make its use more likely as costs would be reduced. Other participants reported that they would use plant treatments only if they had no other option.

### Origins and transmission of knowledge of plant-based medicine

Most knowledge regarding plant-based treatments was passed on via word of mouth, generally through the male family line. However, some female participants reported having been shown by their fathers how to prepare specific remedies.

…“he learnt from his father and he will teach his next generation to his children just his family. It is a business and he gets payment from people who use these remedies. His father told him not to charge too much as otherwise it may not work”…

Generally, people learnt through active participation accompanying their father to collect the plants and assisting with the preparation process. One participant described being able to tell that the preparation of ‘Grawa’ was correct due to the ‘taste’ of the mixture. Plant preparations named by traditional healers were kept within their family only and their recipes guarded and not reported here. There were elements of tradition, religion and superstition surrounding the preparation and efficacy of plant preparations and, occasionally, these were sold for a small fee contributing to the household income. In some cases, participants reported they would not pass on some or all of the information in future as clinics were readily available in the area, or that they believed that some of the plant preparations did not work.

…“there is no transmission of the knowledge through their children…as this current generation don’t want to use those traditional medicines that’s why it is not transmitted to their children so everyone converts to modern medicine”…

A common theme raised by participants was the concept of plant-based medicines as a “*past trend”* and something that people were moving away from in favour of “*modern”* medicine. This was particularly apparent in the younger generation who often reported little interest in learning about plant-based therapies from their elders.

…“because they relate it to old-style religion so they don’t want to know and also modern medicine is available”…

## Discussion

General knowledge of traditional medicines (TM) used as anthelmintics was high in this study, with all groups naming plants used in livestock species generally and 22/29 groups specifically naming plants for use in donkeys. In total, 21 different plant preparations were named for use in livestock. This is in line with previous reports of the wide use of TM’s in developing countries, often attributable to their accessibility and affordability. In Africa, up to 80% of the population use TM to help meet their health care needs [14] and natural plant-derived products have been known for many decades to possess anthelmintic properties [28]. However, many of the previous studies report the use of plant-based anthelmintics for humans [15] or for ruminants, pigs and poultry livestock [16,18,20,22,23]. Further, the majority of evidence for plant-based anthelmintics is in the form of observations rather than controlled studies [28]. To the authors’ knowledge there are no published studies of the use of these materials in equids, despite their immense value to communities in developing countries [29].

Of all the plants named by participants, *V. amygdalina,* was the highest ranked plant for efficacy against gastrointestinal parasites. It is a perennial shrub that is abundant in tropical Africa, including the regions of interest in Ethiopia. It has been used for centuries by humans for the treatment of multiple ailments, and recent research has identified that it may have a number of health benefits such as antimalarial, antimicrobial, antifungal, antitumor, and anti-diabetic effects [30]. There have also been several studies demonstrating its potential as an anthelmintic. For example; a study in puppies in Nigeria demonstrated a significant anthelmintic effect of the aqueous extract of *V. amygdalina* leaves against *Toxocara canis* and *Ancyclostoma caninum *[31] and the aqueous extract of *V. amygdalina* leaves has been shown to reduce faecal egg counts in calves infected with mixed gastrointestinal nematodes by 59.5% [32]. One study investigated the bio-activity of a related species, *V. anthelmintica* and demonstrated a faecal egg count reduction of 73.9% when sheep were administered 3 g/kg of crude aqueous extract of the seeds [33]. *Hagenia abyssinica*, also ranked highly, has well known anti-cestodal properties [15,34] and was reportedly frequently used to treat human infection with tapeworm. Although *H. abyssinica* and *V. amygdalina* were the most frequently named plants here, there were important issues raised about the potential side effects of these two remedies, which ranged in severity and reportedly could include death of the animal if not used correctly. Negative side effects in humans have also been reported with the use of *H. abyssinica.* The most common of these are diarrhoea and abdominal pain. Blindness, changes to the central nervous system, abortion and death, have also been associated with ingestion of a high dose of *H. abyssinica *[15,35]*.*

Amongst the other highly ranked plants, there are reports in the literature of anthelmintic activities, although the evidence is not as compelling as for *V. amygdalina. Withania somnifera* has been identified in previous surveys of ethnoveterinary plants [36] and an *in vitro* study assessing the effect of aqueous extracts of this plant against *Pheretima posthuma* (earthworm) showed a significant effect [37]*. Cucumis prophetarum* has also been identified in previous surveys [38,39]. There are no studies assessing the specific anthelmintic activity of this plant species, however plants in the same family, *Cucurbitaceae,* have been used for centuries as taenicidals and a recent study showed a related species *Cucurbita moschata* to be effective against nematodes *in vitro *[40]. The use of *E. schimperi* and evidence for efficacy *in vivo* and *in vitro* is restricted to taenicidal activity [41]. The informant consensus of plants named in this study was relatively high (close to 1) and indicates good homogeneity of cultural knowledge on the use of plants in the treatment of gastrointestinal parasitic disease suggesting that knowledge is shared between communities. This score indicates that relatively few different taxa of plants were reported by the different groups which may suggest that some of these plants could be efficacious. It was not possible to identify the species of plant that was referred to as ‘Abdul salim’ as the plant itself did not grow in the areas where the study was conducted and, therefore samples could not be collected for specific identification.

The most frequently reported signs associated with gastrointestinal parasites in donkeys were observation of worms in the faeces and loss of body condition. This may indicate a relatively high burden of parasitism prior to any treatment being given. Indeed, previous studies have reported a high prevalence of parasitism within the donkey population in Ethiopia [1,9]. ‘Loss of body condition’ in a donkey is not necessarily pathognomonic for helminthiasis and it was acknowledged by participants that the same plant based preparations were often used for multiple clinical presentations. This may result in some mis-classification bias within this study however ongoing work is investigating a selected number of highly ranked plants for their bio-activity against *Cyathostome spp. in-vitro*.

The preparation methods described were relatively straight forward and often used leaves or whole plants crushed and mixed with water to make an infusion which was then administered. This is akin to preparation methods reported in other ethnoveterinary medicine studies [42,43]. However between groups there was a variety of methods of preparation and measures of ingredients used resulting in limited useful information relating to how these plants are prepared for use in donkeys. It may be that each family group has slightly different preparation methods or may be indicative of a certain amount of ‘trial and error’ involved when extending the use of plant-based medicines traditionally established for use in cattle/small ruminants to donkeys as was reported within this study.

There was no report of prophylactic dosing with anthelmintics among donkeys, and animals were treated based on the recognition of clinical signs. This may, in part, be due to socio-economic pressures influencing the frequency with which medical interventions are sought for donkeys. Further research is required in order to describe how socio-economic and other contextual determinants drive owner decision making regarding preventive health care, particularly given the large population of donkeys within these communities.

Although traditional medicines continue to play a significant role within the community health care system in Ethiopia [44] there appears to be a general shift away from traditional remedies for anthelmintic treatment in this study area due to the availability of clinical services. The perception was that clinics provide a more accurate diagnosis and dosage of medicines and that these represent a modernisation and improvement in practice and had fewer side effects. This finding was similar to that reported by Bussman *et al.* in 2011 [45]. It appears that the younger generation in particular are not as interested in learning about and retaining the knowledge relating to plant-based medicines. Others have shown that ethnoveterinary knowledge is greater in older informants and those with lower education levels [46]. As knowledge of plant-based remedies is passed on through word of mouth and generally stays within family lines, it may be prudent to collect further information for documentation of additional plant-based remedies for use in veterinary species before this information disappears.

There appears to be a widespread practice of drenching donkeys with plant-based remedies for treating many conditions and this poses a significant risk of aspiration pneumonia (cases are regularly reported to the Donkey Sanctuary clinic, *pers comm*.). Additionally, chemical anthelmintics were often reported to be in tablet form which were mixed with water and drenched. Practical alternatives for these problems need to be developed and communicated in order to reduce this risk.

In many cases, people were unable to name the chemical anthelmintic product they used but those that did spoke of “Albendazole”. It is unknown whether the helminth population is susceptible to the treatments available in these regions and whether the method, and dose, given to donkeys is sufficient for control. In other parts of the world a range of anthelmintics (benzimidazoles, tetrahydropyrimidines and macrocyclic lactones) have been widely used against equine gastrointestinal nematodes for many years; however, anthelmintic resistance is present in many populations in developed countries and threatens sustainable control in future [11]. Although the degree of anthelmintic resistance has not yet been established in nematodes of donkeys or horses in Ethiopia, continued use of a limited range of chemical anthelmintics, combined with the effects of under or inappropriate dosing, or inferior quality generic products, are all risk factors for promoting anthelmintic resistance. Benzimidazole resistance in small ruminant nematodes has already been demonstrated in Ethiopia [47,48].

Some potential biases may have been introduced due to the roles of the researchers as veterinarians and animal health assistants. This may have influenced the participants’ discussions to favour clinical medicine; however, it was considered that a good range of views relating to the benefits and disadvantages of both clinic-based medicine and traditional medicine were obtained so we consider this bias to be minimal. The timing of the study was at the beginning of the wet season and consequently, in some regions there was little vegetation evident. This may result in an amount of recall bias among participants leading to some ‘out of season’ plants being omitted from the discussion however, we asked the question about plant use in general and given that people recalled annual trends in plant use it is anticipated that this source of bias would be minimal. There may have been some selection bias with more knowledgeable participants selected by the DA’s who were influential in selecting the participants; however, they were briefed on which participants to recruit and it is believed that we communicated with a broad range of donkey owners.

Further research is required to determine helminth sensitivity to anthelmintic preparations commonly used in these regions. Additionally, further work is warranted to investigate the potential use of plant-based preparations as anthelmintics in donkeys and other species.

Aside from investigating options to overcome the threat of resistance problems, there are several other advantages of using plant-based anthelmintics in developing countries including cost, availability and environmental aspects [28].

## Conclusions

There was extensive knowledge of plant-based treatments for gastrointestinal parasites in donkeys and other livestock in this region in Ethiopia. In donkeys, Koso (*H. abyssinica*) Grawa (*V. amygdalina*), Enkoko (*E. shimperi*) and mixed roots and leaves were the most frequently named and/or highest ranked plants with reported efficacy against gastrointestinal parasites in this species. However, as there appears to be a general shift away from the use of traditional plant-based remedies in the treatment of gastrointestinal parasites in animals it may be prudent to collect further information for documentation of additional plant-based remedies before this knowledge wanes. Results from this study have been triangulated with published research to guide selection of plants for *in vitro* testing against cyathostomins. This may, in future, lead to the identification of an efficacious plant-based remedy that is easily available and readily grown in Ethiopia.

## Methods

### Study area and participants

**Figure 1:**
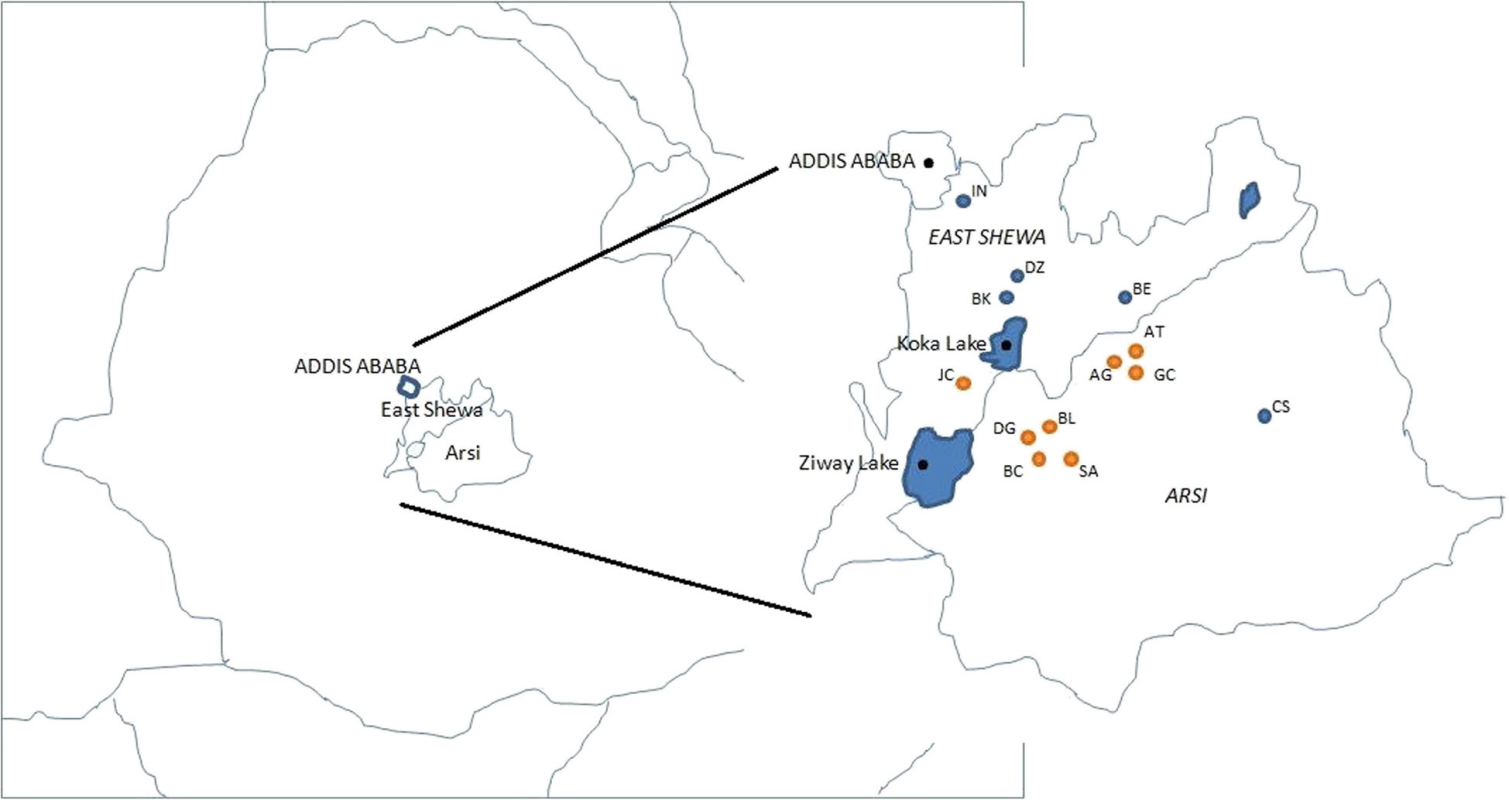
**Map showing East Shewa and Arsi zones of Ethiopia and the Kebeles where the focus groups were held.** Footnote: Two focus group locations are omitted from this map as no longitude/latitude co-ordinates were available. Legend: The map on the left depicts the East Shewa and Arsi zones within Ethiopia. The map on the right shows the kebeles within which the focus groups were held. Blue map points = Donkey sanctuary mobile clinic sites, Orange map points = Areas where donkey sanctuary have not previously accessed. Kebeles are represented by the following initials; BE Buricha (Boset), BK Bekejo, CS Chole Sonkole, DZ Debre Zeyit, IN Insilale, AG Alelu Gasala (Sire), AT Amola Tebo (Sire), BC Boru Cilalo, BL Boru Lencha, DG Dawe Guticha, JC Jawi Cilalo, GC Gasala Chicha, SA Sibu Abidir.

The study area consisted of 15 kebeles in the Eastern Shewa and Arsi zones of the Oromia region of Ethiopia (Figure 1). Villages were purposively selected for their varied topography and logistical accessibility and included highland, mid-highland and lowland sites. Six villages had been previously exposed to an equine non-governmental organization (NGO) (which included equine education programmes and clinical services) whilst 9 were unexposed to these services. Data collection was conducted over a 6-week period during June and July 2011, representing the beginning of the wet season in this region of Ethiopia. Participants were eligible for inclusion if they owned a donkey. Participation was entirely voluntary and owners were free to leave at any point. Participants were recruited using local development agents (DA) assigned from the relevant Bureau of Agriculture. Each DA was briefed about the study and asked to select donkey owners of a range of ages (>16 years of age). Two groups per village (with the exception of one where only one group was conducted), with 6–8 people per group, were recruited. The study was conducted in the local language (Afan Oromo/Amharic) and was facilitated by the author (CS) and an Ethiopian animal health worker (AHW) (GT) who acted as co-facilitator and translator. Both had received previous training in participatory approaches and were experienced in facilitating focus group discussions. Participatory methods used in this study were based upon previously published participatory and ethnobotanical study designs [27,49,50].

### Focus group discussions

At the beginning of each focus group, GT gave an overview of the study and affiliated research groups and verbal informed consent was gained. The group interviews were semi-structured, including some key questions with opportunities for open discussion and utilised a number of different participatory methodologies (Table 5). Each group participated in constructing 2 sets of matrices, which were drawn on laminated white card and photographed. The first key question was, “which animals / species do you consider are affected by worms / gastrointestinal parasites?” This generated the first row of the matrix labelling animal species that were named by the group. A matrix of signs in each animal species was constructed by asking, “how do you know that an animal has worms-what signs do you see?” Open discussions relating to ‘signs of worms’, was investigated further with probing questions to explore the nature of worms seen.

**Table 5:** Focus group format: key questions and group tasks initiated within each focus group discussion

**Key question**	**Participatory activity**
Which animals / species do you consider are affected by worms / gastrointestinal parasites?	List responses
How do you know that an animal has worms? What signs do you see?	List responses
	Construct matrix of species and signs of worms
What do you use to treat worms in your donkey / cattle and small ruminant? (List and then rank by preference / efficacy within each species)	List treatments
	Rank by preference / efficacy within each species
Why do you use these plants?	Matrix and group discussion
A matrix was constructed with each plant species named and matrix headings were cost, ease of use, availability, side effects, when particular worm seen, animal species treated and benefits working ability.	
The matrix was filled in with + / - or neutral for each column.	
Additional questions were asked during discussions:	
Where do you get the plant from?	
How do you know that it is effective?	
If plant based remedies not volunteered for worms–why not?	
Do you or anyone in your village use plant based treatments for anything else?	
Are there any circumstances where you would return to the use of plant based treatments?	
Would you spend money on worming your donkeys?	Group discussion
If you thought a plant was effective against worms would you grow it specifically to use in your donkeys?	Group discussion
Are there people in the village that know about or supply plant based treatments for wormers?	Group discussion

The next key question was, “what do you do to treat worms in these animals?” Responses were filled in alongside the listed signs and under the appropriate species.

The second matrix focused upon plant-based medicines named for use in donkeys within the first matrix. The names of the volunteered plants were listed along one side of the matrix and for each plant, brief details of where the plant/root or seed was obtained, how owners knew that this was an effective treatment and any side effects were recorded. If more than one plant was named for use in donkeys, the group were asked to arrive at a consensus of how these were ranked in terms of effectiveness. Photographs were taken of the matrices from each group.

During the course of the discussion, further questions were asked to explore general opinions relating to plant-based medicines and the importance of deworming donkeys. Care was taken not to influence the content of the discussion with leading questions. Open-ended questions were used to encourage discussion and exploration of the topic. To optimise data quality, all responses from participants were volunteered and participants were encouraged to contribute freely to the discussion by the facilitators.

### Data management

The discussions were translated into English and recorded on a digital Dictaphone and this was then transcribed. Excel software was used to tabulate the photographed matrices. A list of plants named for donkeys was generated including frequency tables of how many groups named each plant type and ranking data to show the perceived effectiveness of the plants for use in donkeys. An average ranking for each plant was produced (by summing the rank positions and dividing by the total number of groups identifying that plant) along with a combined rank score. This was calculated by re-assigning the ranks with a score. For example, if a group named 4 plants for use in donkeys then the plant they ranked 1st and most effective was given a score of 4, the 2nd a score of 3 etc. Subsequently, the total scores for each plant from each group were added together to give a combined rank score. An estimation of the variability and homogeneity of knowledge of plants used to treat gastrointestinal parasites was determined using the Informant consensus factor [51]. This was calculated based on [52] as follows:

$$F_{\mathrm{rc}}=\left(n_{\mathrm{ur}}-n_{t}/n_{\mathrm{ur}}-1\right)$$

Where n_ur_ was the number of usage reports (or in this case groups naming the plant for use in donkeys) and n_t_ was the number of taxa used (or named by the groups, excluding mixed preparations). Recent studies have used informant consensus as a means to examine cultural knowledge and diversity of use of plant species for different clinical conditions [38,53].

A thematic analysis [54,55] of the content of the translated discussion relevant to participants’ current attitudes and beliefs surrounding traditional plant-based remedies for gastrointestinal parasites was conducted with the aid of NVivo 8 data handling software. This involved reading all transcripts to become familiar with the data, sorting quotes discussing similar aspects relating to plant-based medicine into themes, reviewing the grouped themes and summarising the concepts. This facilitated the analysis and summarisation of the variety of responses relating to the key questions.

### Plant specimens and identification

Cutting samples were collected for each plant named in the focus groups including (where possible) the leaves, stems, flowers or seeds [56]. Where the plant was unknown to the research team, the participant was asked to show an example of the plant if it was locally available. Photographs were also taken of each plant to include close up pictures of the leaf / branch structure and any flowers or seed heads. A note was made of the date and site of collection. All plants were dried and pressed in preparation for formal identification at the National Herbarium, Addis Ababa University. Subsequent to identification of plant species, the results were triangulated with available literature to investigate evidence of bio-activity against gastrointestinal parasites in any species.

This study was reviewed and received ethical approval from the University of Liverpool research ethics committee and the University of Addis Ababa College of Veterinary Medicine and Agriculture.

## Competing interests

The authors declare that they have no competing interests.

## Authors' contributions

GP, JH and JM conceived the study and acquired the funding. All authors contributed to the study design. CS, LP and GT coordinated the study and collected all the data. CS performed all data analysis. GP drafted the manuscript with major input by CS. All authors were involved in revising the manuscript and approved the final manuscript.
